# Supplementary material for: Distinct diet-microbiome associations in autism spectrum disorder
Source: Nat Commun. 2025 Dec 31;17:3109. doi: 10.1038/s41467-025-67711-7 (PMC13039903; doi:10.1038/s41467-025-67711-7)
Supplement: Supplementary file 1 — Supplementary Information [file 41467_2025_67711_MOESM1_ESM.pdf]

Supplementary information

Supplementary Fig. 1: Diet-microbiome signatures correlations in children with and without autism spectrum disorder (ASD).

Supplementary Fig. 2: Autism spectrum disorder (ASD)-specific diet-microbiome functional associations.

Supplementary Fig. 3: Autism spectrum disorder (ASD)-specific associations between dietary metrics and microbial functional KEGG orthologies.

Supplementary Fig. 4: Interaction effects of autism spectrum disorder (ASD) status on diet-microbiome associations.

Supplementary Fig. 5: Predictions for dietary profiling based on microbial function.

Supplementary Fig. 6: Predicted versus actual dietary value correlations from gut microbiome-derived machine learning models.

Supplementary Fig. 7: Attenuated dietary responsiveness of the gut microbiome and the mechanistic links in children without autism spectrum disorder (ASD).

Supplementary Fig. 8: Significant mediating role of specific microbial species in the associations between dietary metrics and core symptoms of children with autism spectrum disorder (ASD).

Supplementary Fig. 9: Stability of microbial ecological networks under low emulsifier exposure.

Supplementary Fig. 10: Autism spectrum disorder (ASD)-linked microbial signatures independent of diet and medication.

Supplementary Fig. 11: Effect modifications of gastrointestinal (GI) complications on the diet-microbiome associations in children with autism spectrum disorder (ASD) and those without.

Supplementary Table S1: Dietary summary of participants in the study based on the tertiles of the Chinese Children Healthy Dietary Index (CCDI).

Supplementary Table S2: Relationships between various dietary indices and gut microbial features specific to autism spectrum disorder (ASD) and the effect modification of ASD across the study population.

Supplementary Table S3: Sensitivity analysis of diet–microbiome correlations in a 1:1 matched cohort of children with and without autism spectrum disorder (ASD).

Supplementary Table S4: Comparisons of demographic factors and dietary assessments between individuals with and without autism spectrum disorder (ASD).

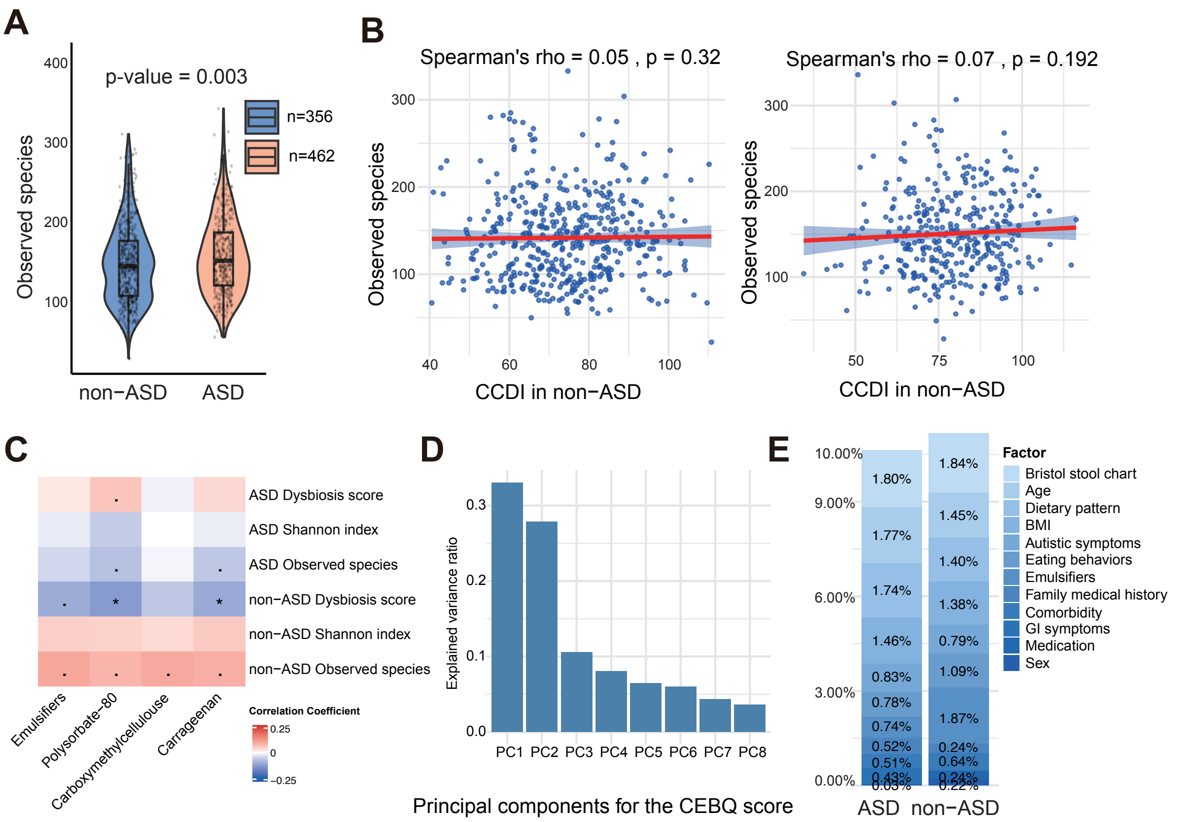

Supplementary Fig. 1: Diet-microbiome signatures correlations in children with and without autism spectrum disorder (ASD). (A) Comparison of alpha diversity characterized by observed species between ASD and non- ASD peers. (B) The Chinese Children Healthy Dietary Index (CCDI) correlations with observed species in ASD and non-ASD children. (C) Correlations heatmap of synthesized-emulsifier exposures with gut microbial features. (D) Principle component (PC) loadings for the Children's Eating Behavior Questionnaire (CEBQ) subscales. (D) The effect size of diet and other confounders on the variability of microbial functional pathways. The proportion of gut microbial variation explained by various phenotype factors was assessed using PERMANOVA. Bray-Curtis distance matrices, based on the relative abundances of microbial species, were analyzed using the adonis function in the R package vegan with 9,999 permutations.

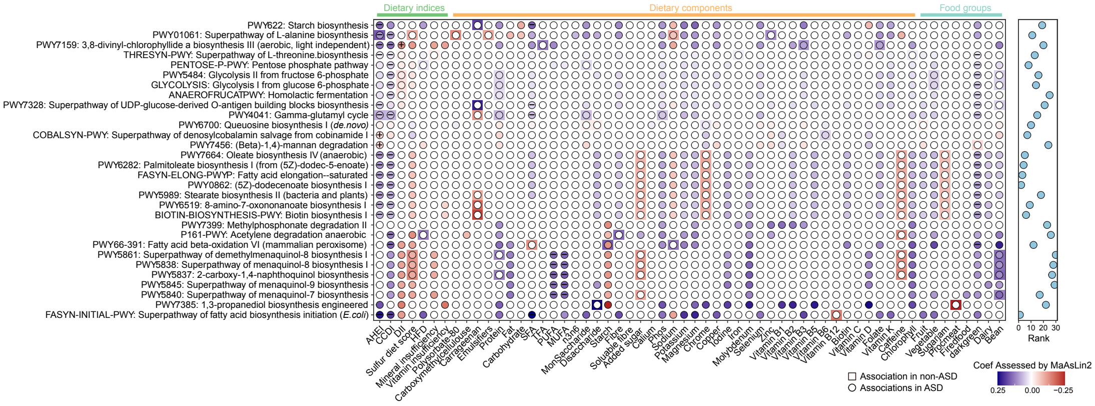

Supplementary Fig. 2: Autism spectrum disorder (ASD)-specific diet-microbiome functional associations. Heatmap displays the top 30 diet-associated microbial functional pathways in children with ASD (in circle shape) that lost significant associations in non-ASD peers (in rectangular shape). Significant associations (MaAsLin2, qval < 0.1, adjusted for age, gender, and gastrointestinal conditions) are color-coded by dietary category: green (dietary indices), orange (nutritional components), and blue (food groups). The significant associations (qval < 0.05) are annotated with + or - according to the directions of associations.

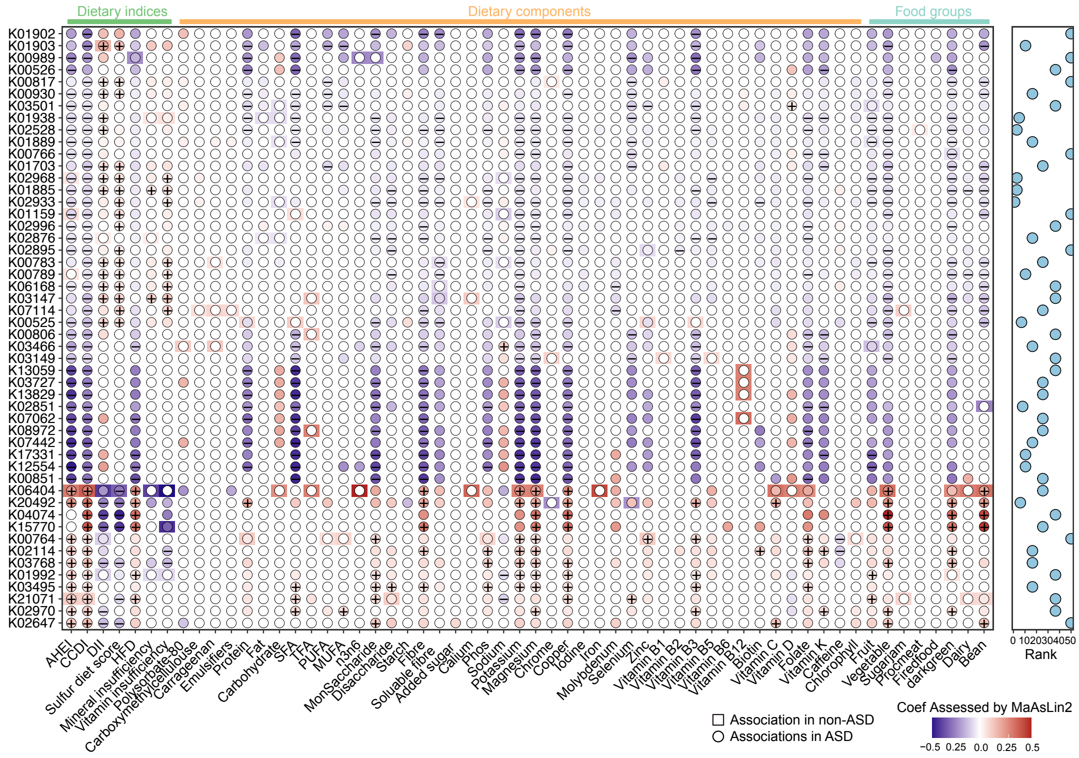

Supplementary Fig. 3: Autism spectrum disorder (ASD)-specific associations between dietary metrics and microbial functional KEGG orthologies. Heatmap displays the top 50 diet-associated microbial functional genes in children with ASD (in circle shape) that lost significant associations in non-ASD peers (in rectangular shape). Significant associations (MaAsLin2, qval < 0.1, adjusted for age, gender, and gastrointestinal conditions) are color-coded by dietary category: green (dietary indices), orange (nutritional components), and blue (food groups). The significant associations (qval < 0.05) are annotated with + or - according to the directions of associations.

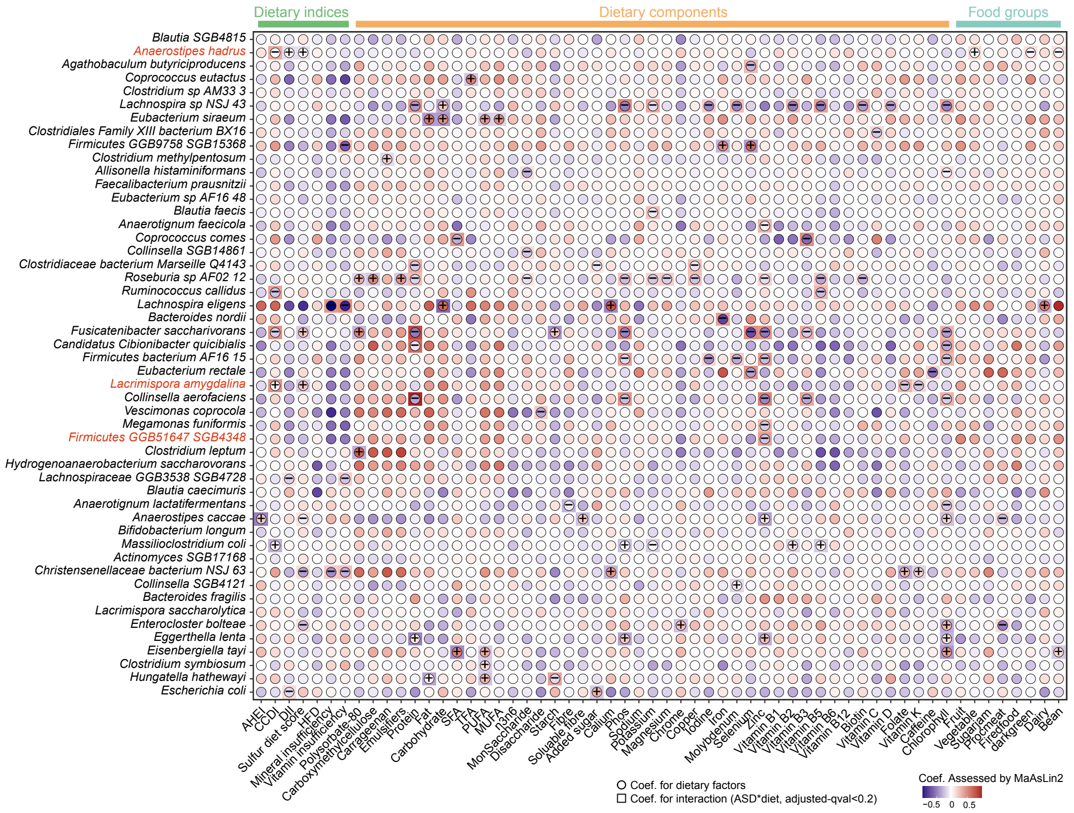

Supplementary Fig. 4: Interaction effects of autism spectrum disorder (ASD) status on diet-microbiome associations. Heatmap displays the identified diet-associated microbial species and their association coefficients with dietary metrics. Coefficients derived from the pooled sample (children with and without ASD) are indicated by circles, and significant coefficients for the ASD-by-diet interaction term are indicated by rectangular. Significant interactions (qval < 0.2) are marked with + or - corresponding to the effect direction (see Supplementary Table S5 for details). Analyses were adjusted for age, sex, and gastrointestinal conditions using MaAsLin2.

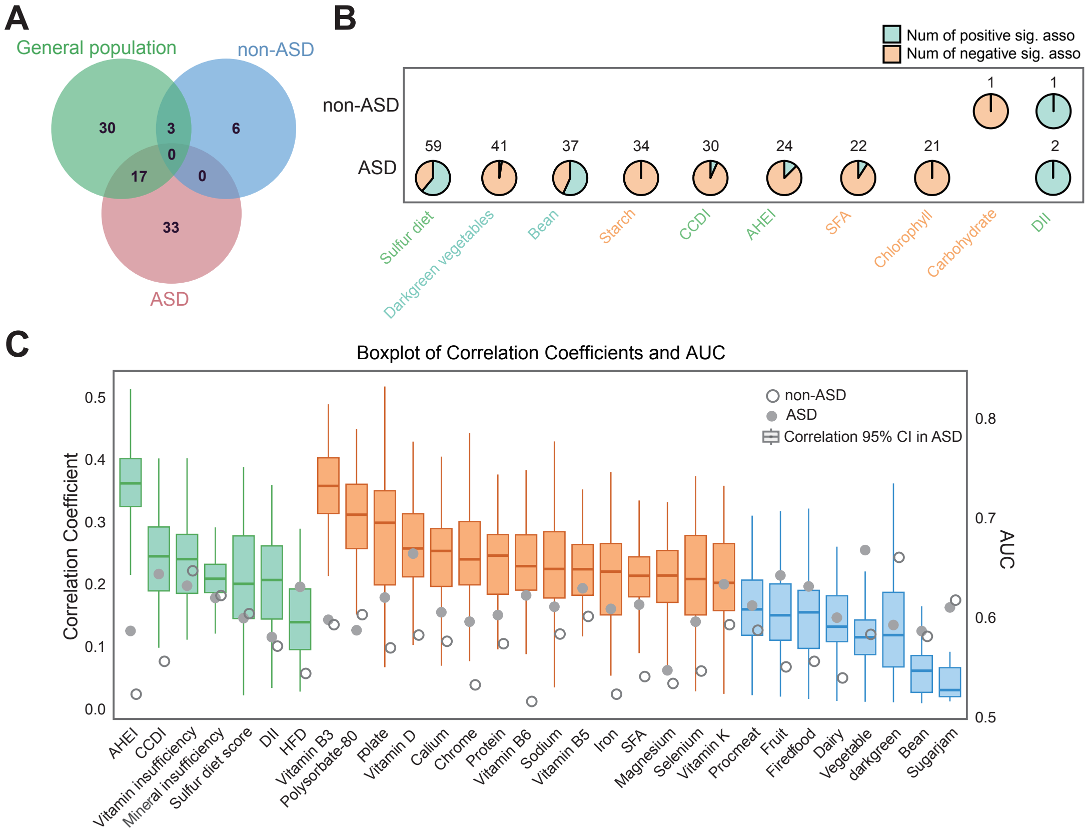

Supplementary Fig. 5: Predictions for dietary profiling based on microbial function. (A) Venn diagram identifies overlapping and distinct diet-functional microbiome associations between autism spectrum disorder (ASD) and non-ASD peers. (B) Sector diagrams quantify the prominence of significant diet-microbial function associations (top: non-ASD; bottom: ASD). (C) Machine learning models demonstrate the predictive power of microbial function by generating correlation results (left axis) and AUC values (right axis), respectively. Box plots display the agreement between actual dietary values and microbial function-based predictions from 10-fold cross-validated regression models in ASD, with error bars representing the 95% confidence interval (CI). Predictive performance is further quantified by median AUC values from binary classification (solid circles: ASD; hollow circles: non-ASD).

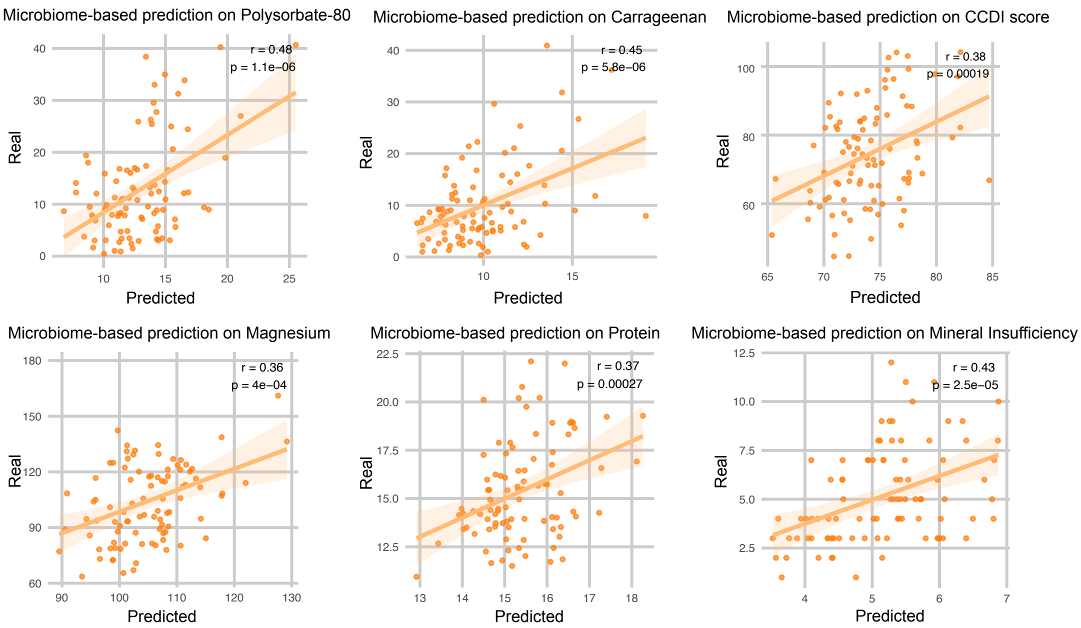

Supplementary Fig. 6: Predicted versus actual dietary value correlations from gut microbiome-derived machine learning models.

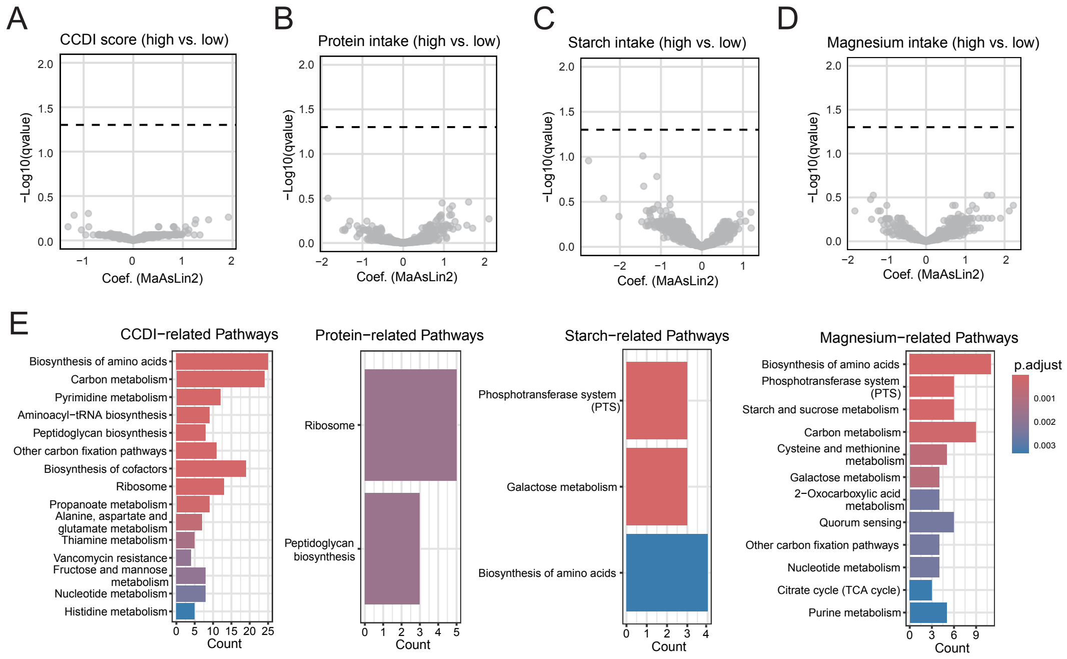

Supplementary Fig. 7: Attenuated dietary responsiveness of the gut microbiome and the mechanistic links in children without autism spectrum disorder (ASD). Microbial response to dietary factors: the Chinese Children Dietary Index (CCDI) (A), protein (B), magnesium (C), and starch (D), in non-autism spectrum disorder (ASD) peers shows limited associations (MaAsLin2; qval > 0.1) after covariate adjustment. Coefficients compare high vs. low intake groups (reference). (E) KEGG orthology (KO) enrichment analysis for dietary associations, inducing CCDI, protein, magnesium, and starch intake.

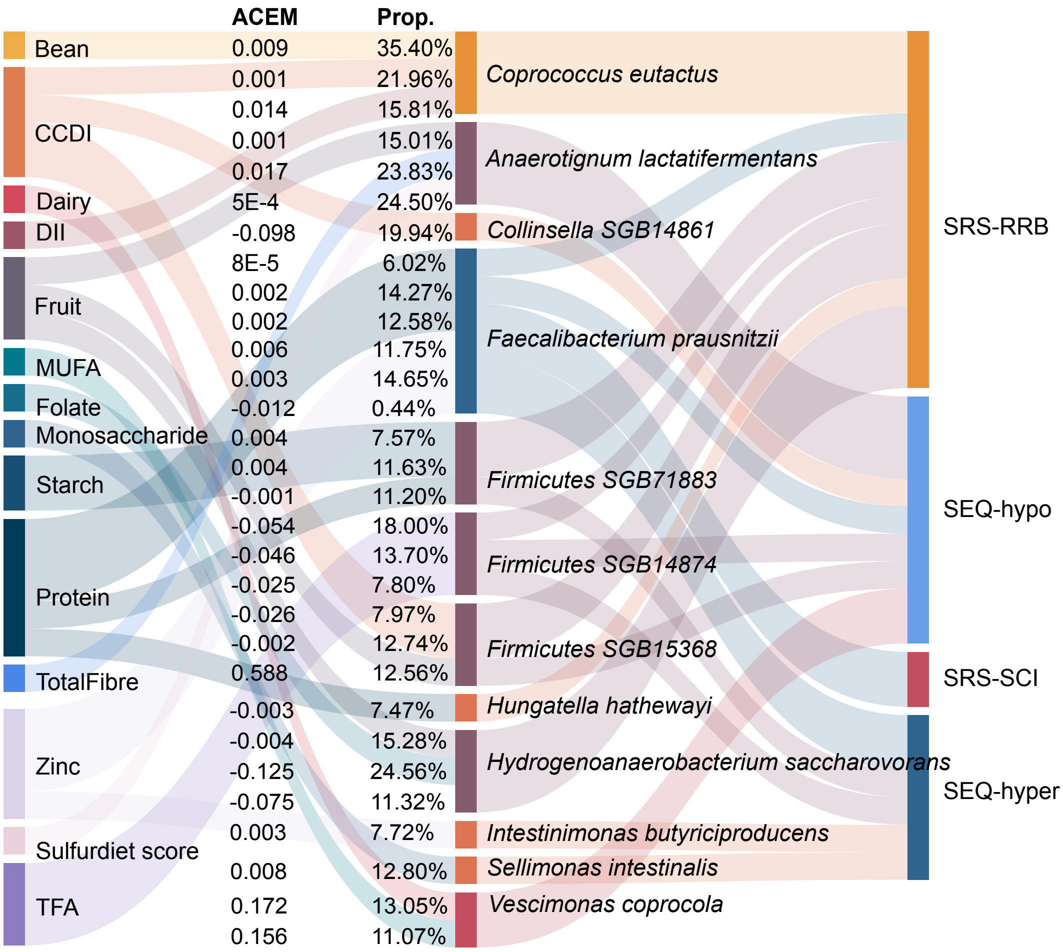

Supplementary Fig. 8: Significant mediating role of specific microbial species in the associations between dietary metrics and core symptoms of children with autism spectrum disorder (ASD). Details were provided in Supplementary Table S11. ACEM: average causal mediation effect; SRS, the Social Responsiveness Scale; SEQ, the Sensory experiences questionnaire; CCDI, the Chinese Children Healthy Dietary Index; DII, Dietary Inflammatory Index; MUFA, monounsaturated fatty acids; TFA, trans-fatty acids.

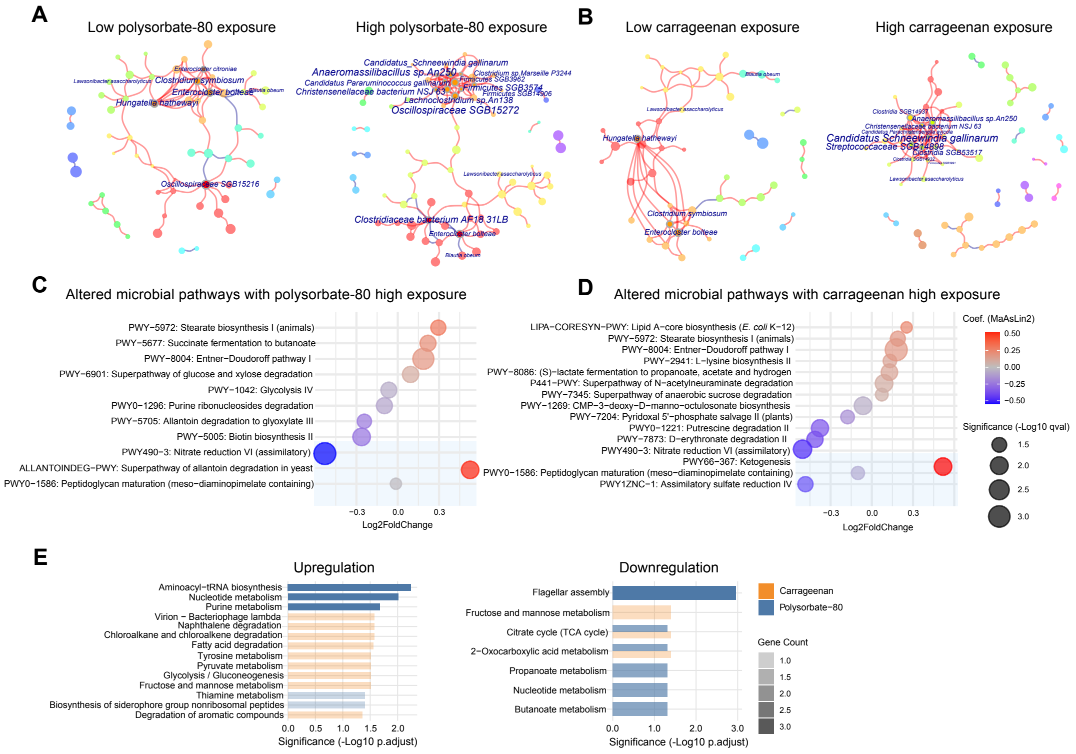

Supplementary Fig. 9: Stability of microbial ecological networks under low emulsifier exposure. (A-B) Preserved microbial co-occurrence networks in autism spectrum disorder (ASD) (right) and neurotypical (left) children with low exposures to polysorbate-80 (A) and carrageenan (B). (C-D) Functional pathway modulation. Changes in microbial metabolic pathways (MetaCyc) in responses to polysorbate-80 (C) and carrageenan (D). Analyzed pathways had >10% sample prevalence. Significant associations (qval < 0.1) are color-coded (red: enrichment in high intake; blue: depletion). (E) KEGG orthology (KO) enrichment analysis of downregulated functional signals associated with high exposure to polysorbate-80/carrageenan exposure.

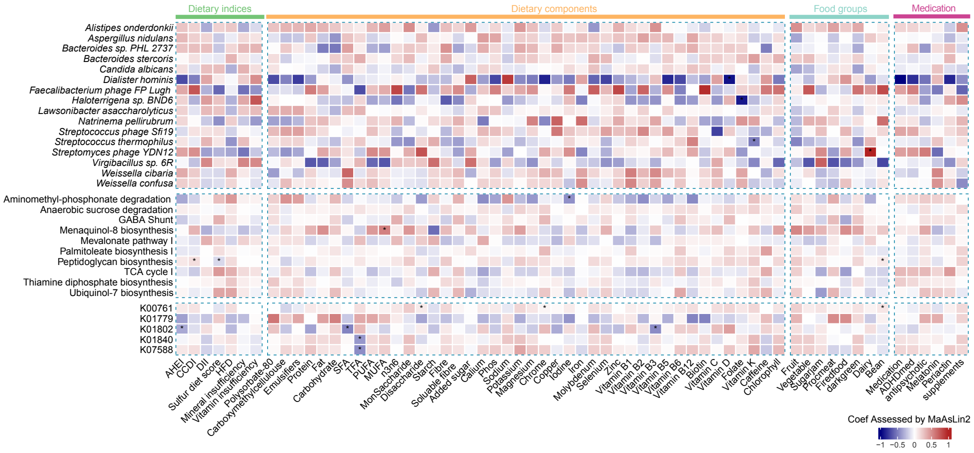

Supplementary Fig. 10: Autism spectrum disorder (ASD)-linked microbial signatures independent of diet and medication. Asterisks indicate significant associations (qval < 0.1, adjusted for age, gender, and gastrointestinal conditions) in line with the diet-microbiome association analysis strategy using MaAsLin2.

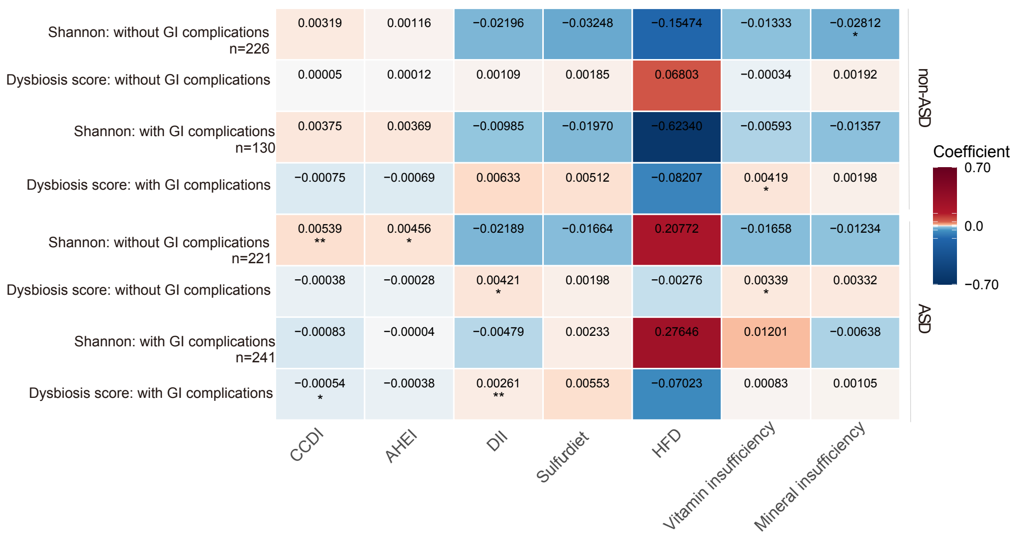

Supplementary Fig. 11: Effect modifications of gastrointestinal (GI) complications on the diet-microbiome associations in children with autism spectrum disorder (ASD) and those without.

Supplementary Table S1: Dietary summary of participants in the study based on the tertiles of the Chinese Children Healthy Dietary Index (CCDI).

	**CCDI (Tertile 1) N=273**	**CCDI (Tertile 2) N=273**	**CCDI (Tertile 3) N=272**	**p-value**
Dietary indices				
AHEI	46 (39, 54)	54 (46, 63)	60 (55, 70)	<0.001
DII	1.36 (0.38, 2.57)	0.19 (-0.98, 1.55)	-1.58 (-2.65, -0.51)	<0.001
Sulfur-diet score	0.84 (0.28, 1.39)	0.19 (-0.41, 0.72)	-0.54 (-1.13, 0.05)	<0.001
HFD	0.42 (0.38, 0.47)	0.48 (0.42, 0.52)	0.50 (0.46, 0.55)	<0.001
Mineral insufficiency	6.00 (4.00, 8.00)	5.00 (4.00, 7.00)	3.00 (2.00, 5.00)	<0.001
Vitamin insufficiency	9.00 (7.00, 10.00)	7.00 (5.00, 9.00)	5.00 (3.00, 6.00)	<0.001
Dietary components				
Total energy (kcal)	1,761 (1,393, 2,177)	1,716 (1,328, 2,274)	1,973 (1,613, 2,451)	<0.001
Protein (%en)	14.21 (12.85, 16.21)	15.42 (14.04, 16.99)	16.94 (15.38, 18.81)	<0.001
Fat (%en)	30.6 (27.2, 34.0)	30.1 (27.0, 33.3)	30.4 (27.3, 32.7)	0.300
Carbohydrate (%en)	55 (50, 59)	54 (51, 58)	53 (50, 57)	0.010
Starch (g/1000kcal)	78 (65, 97)	78 (65, 90)	71 (60, 83)	<0.001
Total Fibre (g/1000kcal)	5.42 (4.54, 6.26)	6.40 (5.69, 7.33)	7.60 (6.68, 8.73)	<0.001
Food groups				
Fruit (g/1000kcal)	78 (43, 124)	123 (73, 195)	186 (133, 258)	<0.001
Vegetables (g/1000kcal)	85 (46, 131)	154 (113, 212)	284 (198, 381)	<0.001
Sugar/jam (g/1000kcal)	5 (1, 11)	5 (1, 10)	4 (1, 11)	0.990
Processed-meat (g/1000kcal)	19 (11, 40)	18 (9, 33)	18 (8, 31)	0.130
Fired food (g/1000kcal)	40 (24, 63)	36 (19, 58)	37 (21, 60)	0.300
Dark green vegetables (g/1000kcal)	11 (4, 26)	23 (9, 40)	40 (23, 62)	<0.001
Dairy (g/1000kcal)	39 (4, 111)	64 (24, 131)	133 (63, 236)	<0.001
Bean (g/1000kcal)	3 (0, 14)	18 (10, 31)	40 (23, 59)	<0.001
Emulsifiers				
Polysorbate-80 (g/kg bw)	7.6 (4.7, 11.5)	7.0 (4.2, 10.8)	6.8 (4.1, 10.4)	0.045
Carboxymethylcellulouse (g/kg bw)	9 (6, 16)	9 (5, 14)	9 (6, 13)	0.200
Carrageenan (g/kg bw)	15 (8, 25)	13 (8, 22)	14 (8, 22)	0.200

The study populations were categorized into tertiles of the Chinese Children Healthy Dietary Index (CCDI), indicating a less healthy dietary pattern with an average score of 63 (57, 67), a moderate quality with an average score of 76 (73, 80), and a healthier pattern with an average score of 91 (86, 96). Group comparisons were performed using Kruskal-Wallis rank sum tests for continuous variables and Pearson's Chi-squared tests were used for categorical variables. All reported exact p-values are two-tailed testing. More details have been provided in the methods. AHEI, alternative healthy eating index; DII, dietary inflammatory index; HFD, healthy food diversity.

Supplementary Table S2: Relationships between various dietary indices and gut microbial features specific to autism spectrum disorder (ASD) and the effect modification of ASD across the study population.

	ASD (n=462)		non-ASD (n=356)		general population (n=818)		
**CCDI**							
**Spearman correlation**	ρ coefficient	p-value	ρ coefficient	p-value	ρ coefficient	p-value	
Gut dysbiosis score	-0.121	0.009	-0.073	0.167	-0.120	0.001	
Shannon index	0.140	0.003	0.108	0.043	0.108	0.002	
**Kendall correlation**	τ coefficient	p-value	τ coefficient	p-value	τ coefficient	p-value	
Gut dysbiosis score	-0.076	0.078	-0.049	0.351	-0.081	0.001	
Shannon index	0.093	0.049	0.070	0.185	0.073	0.002	
**Pearson correlation**	ρ coefficient	p-value	ρ coefficient	p-value	ρ coefficient	p-value	
Gut dysbiosis score	-0.110	0.020	-0.087	0.099	-0.128	<0.001	
Shannon index	0.130	0.006	0.111	0.037	0.105	0.003	
**Multivariable-adjusted regression model**	β coefficient	p-value	β coefficient	p-value	β coefficient	p-value	p for interaction
Gut dysbiosis score	-0.0004	0.042	-0.0002	0.210	-0.0002	0.244	0.521
Shannon index	0.004	0.010	0.003	0.078	0.003	0.047	0.659
**AHEI**							
**Spearman correlation**	ρ coefficient	p-value	ρ coefficient	p-value	ρ coefficient	p-value	
Gut dysbiosis score	-0.066	0.158	-0.040	0.448	-0.064	0.069	
Shannon index	0.054	0.245	0.064	0.232	0.062	0.077	
**Kendall correlation**	τ coefficient	p-value	τ coefficient	p-value	τ coefficient	p-value	
Gut dysbiosis score	-0.045	0.145	-0.024	0.499	-0.043	0.066	
Shannon index	0.037	0.241	0.042	0.242	0.042	0.075	
**Pearson correlation**	ρ coefficient	p-value	ρ coefficient	p-value	ρ coefficient	p-value	
Gut dysbiosis score	-0.060	0.197	-0.034	0.520	-0.060	0.088	
Shannon index	0.047	0.316	0.047	0.373	0.052	0.138	
**Multivariable-adjusted regression model**	β coefficient	p-value	β coefficient	p-value	β coefficient	p-value	p for interaction

Gut dysbiosis score	-0.0003	0.221	-0.0001	0.541	-0.0002	0.521	0.743
Shannon index	0.002	0.281	0.002	0.393	0.002	0.325	0.971
**DII**							
**Spearman correlation**	ρ coefficient	p-value	ρ coefficient	p-value	ρ coefficient	p-value	
Gut dysbiosis score	0.116	0.012	0.133	0.012	0.136	<0.001	
Shannon index	-0.066	0.156	-0.085	0.109	-0.081	0.020	
**Kendall correlation**	τ coefficient	p-value	τ coefficient	p-value	τ coefficient	p-value	
Gut dysbiosis score	0.079	0.012	0.089	0.012	0.091	<0.001	
Shannon index	-0.045	0.152	-0.058	0.102	-0.055	0.019	
**Pearson correlation**	ρ coefficient	p-value	ρ coefficient	p-value	ρ coefficient	p-value	
Gut dysbiosis score	0.119	0.011	0.126	0.017	0.131	<0.001	
Shannon index	-0.075	0.109	-0.078	0.144	-0.082	0.019	
**Multivariable-adjusted regression model**	β coefficient	p-value	β coefficient	p-value	β coefficient	p-value	p for interaction
Gut dysbiosis score	0.003	0.021	0.003	0.034	0.003	0.061	0.734
Shannon index	-0.013	0.185	-0.014	0.209	-0.015	0.174	0.983
**Sulfur diet score**							
**Spearman correlation**	ρ coefficient	p-value	ρ coefficient	p-value	ρ coefficient	p-value	
Gut dysbiosis score	0.081	0.081	0.073	0.170	0.097	0.005	
Shannon index	-0.007	0.880	-0.100	0.059	-0.060	0.085	
**Kendall correlation**	τ coefficient	p-value	τ coefficient	p-value	τ coefficient	p-value	
Gut dysbiosis score	0.053	0.088	0.049	0.167	0.065	0.006	
Shannon index	-0.004	0.890	-0.065	0.067	-0.040	0.086	
**Pearson correlation**	ρ coefficient	p-value	ρ coefficient	p-value	ρ coefficient	p-value	
Gut dysbiosis score	0.070	0.133	0.077	0.149	0.093	0.007	
Shannon index	-0.012	0.801	-0.076	0.152	-0.051	0.144	
**Multivariable-adjusted regression model**	β coefficient	p-value	β coefficient	p-value	β coefficient	p-value	p for interaction
Gut dysbiosis score	0.003	0.206	0.003	0.245	0.002	0.333	0.831

Shannon index	-0.003	0.853	-0.023	0.234	-0.023	0.240	0.463
**HFD**							
**Spearman correlation**	ρ coefficient	p-value	ρ coefficient	p-value	ρ coefficient	p-value	
Gut dysbiosis score	-0.080	0.087	0.006	0.917	-0.055	0.117	
Shannon index	0.051	0.277	-0.035	0.509	0.018	0.611	
**Kendall correlation**	τ coefficient	p-value	τ coefficient	p-value	τ coefficient	p-value	
Gut dysbiosis score	-0.052	0.092	0.005	0.882	-0.036	0.121	
Shannon index	0.034	0.279	-0.024	0.504	0.012	0.599	
**Pearson correlation**	ρ coefficient	p-value	ρ coefficient	p-value	ρ coefficient	p-value	
Gut dysbiosis score	-0.070	0.133	0.031	0.566	-0.047	0.178	
Shannon index	0.054	0.243	-0.049	0.353	0.019	0.589	
**Multivariable-adjusted regression model**	β coefficient	p-value	β coefficient	p-value	β coefficient	p-value	p for interaction
Gut dysbiosis score	-0.028	0.394	0.020	0.538	0.035	0.339	0.143
Shannon index	0.166	0.491	-0.367	0.196	-0.376	0.180	0.119
**Vitamin Insufficiency**							
**Spearman correlation**	ρ coefficient	p-value	ρ coefficient	p-value	ρ coefficient	p-value	
Gut dysbiosis score	0.013	0.779	0.064	0.232	0.037	0.285	
Shannon index	0.052	0.261	-0.059	0.267	0.003	0.928	
**Kendall correlation**	τ coefficient	p-value	τ coefficient	p-value	τ coefficient	p-value	
Gut dysbiosis score	0.010	0.764	0.046	0.221	0.027	0.278	
Shannon index	0.037	0.257	-0.042	0.257	0.002	0.946	
**Pearson correlation**	ρ coefficient	p-value	ρ coefficient	p-value	ρ coefficient	p-value	
Gut dysbiosis score	0.008	0.857	0.064	0.228	0.030	0.389	
Shannon index	0.057	0.219	-0.050	0.349	0.011	0.745	
**Multivariable-adjusted regression model**	β coefficient	p-value	β coefficient	p-value	β coefficient	p-value	p for interaction
Gut dysbiosis score	0.002	0.123	0.001	0.347	0.002	0.103	0.641
Shannon index	-0.001	0.931	-0.009	0.327	-0.143	0.116	0.181

**Mineral insufficiency**							
**Spearman correlation**	ρ coefficient	p-value	ρ coefficient	p-value	ρ coefficient	p-value	
Gut dysbiosis score	0.030	0.515	0.061	0.254	0.048	0.167	
Shannon index	-0.007	0.888	-0.108	0.042	-0.054	0.121	
**Kendall correlation**	τ coefficient	p-value	τ coefficient	p-value	τ coefficient	p-value	
Gut dysbiosis score	0.021	0.533	0.041	0.272	0.034	0.176	
Shannon index	-0.004	0.901	-0.078	0.039	-0.039	0.115	
**Pearson correlation**	ρ coefficient	p-value	ρ coefficient	p-value	ρ coefficient	p-value	
Gut dysbiosis score	0.015	0.749	0.095	0.073	0.047	0.176	
Shannon index	0.011	0.822	-0.117	0.028	-0.045	0.197	
**Multivariable-adjusted regression model**	β coefficient	p-value	β coefficient	p-value	β coefficient	p-value	p for interaction
Gut dysbiosis score	0.002	0.072	0.002	0.114	0.002	0.044	0.588
Shannon index	-0.010	0.268	-0.021	0.028	-0.025	0.006	0.135

Statistical analysis was performed using Spearman, Kendall's τ, Pearson correlation two-tailed tests, and generalized linear models with adjustments of age, sex, Bristol Stool Chart, GI conditions, and autistic symptoms. All reported exact p-values with significance defined as < 0.05. An interaction term (diet × ASD) was applied in the generalized linear models to determine the effect modification in the general population regardless of ASD status.

Supplementary Table S3: Sensitivity analysis of diet–microbiome correlations in a 1:1 matched cohort of children with and without autism spectrum disorder (ASD).

	ASD (n-356)		non-ASD (n=356)	
**CCDI**				
**Spearman correlation**	ρ coefficient	p-value	ρ coefficient	p-value
Gut dysbiosis score	-0.141	0.008	-0.073	0.167
Shannon index	0.106	0.045	0.108	0.043
**Kendall correlation**	τ coefficient	p-value	τ coefficient	p-value
Gut dysbiosis score	-0.093	0.079	-0.049	0.351
Shannon index	0.071	0.078	0.070	0.185
**Pearson correlation**	ρ coefficient	p-value	ρ coefficient	p-value
Gut dysbiosis score	-0.134	0.011	-0.087	0.099
Shannon index	0.098	0.045	0.111	0.037
**Multivariable-adjusted regression model**	β coefficient	p-value	β coefficient	p-value
Gut dysbiosis score	-0.0004	0.065	-0.0002	0.210
Shannon index	0.003	0.066	0.003	0.078
**AHEI**				
**Spearman correlation**	ρ coefficient	p-value	ρ coefficient	p-value
Gut dysbiosis score	-0.043	0.419	-0.040	0.448
Shannon index	0.030	0.574	0.064	0.232
**Kendall correlation**	τ coefficient	p-value	τ coefficient	p-value
Gut dysbiosis score	-0.030	0.404	-0.024	0.499
Shannon index	0.019	0.585	0.042	0.242
**Pearson correlation**	ρ coefficient	p-value	ρ coefficient	p-value
Gut dysbiosis score	-0.043	0.413	-0.034	0.520
Shannon index	0.029	0.588	0.047	0.373
**Multivariable-adjusted regression model**	β coefficient	p-value	β coefficient	p-value

Gut dysbiosis score	-0.0003	0.290	0.000	0.541
Shannon index	0.003	0.106	0.002	0.393
**DII**				
**Spearman correlation**	ρ coefficient	p-value	ρ coefficient	p-value
Gut dysbiosis score	0.082	0.122	0.133	0.012
Shannon index	-0.067	0.207	-0.085	0.109
**Kendall correlation**	τ coefficient	p-value	τ coefficient	p-value
Gut dysbiosis score	0.056	0.115	0.089	0.012
Shannon index	-0.046	0.199	-0.058	0.102
**Pearson correlation**	ρ coefficient	p-value	ρ coefficient	p-value
Gut dysbiosis score	0.085	0.108	0.126	0.017
Shannon index	-0.077	0.149	-0.078	0.144
**Multivariable-adjusted regression model**	β coefficient	p-value	β coefficient	p-value
Gut dysbiosis score	0.004	0.030	0.003	0.034
Shannon index	-0.016	0.163	-0.014	0.209
**Sulfur diet score**				
**Spearman correlation**	ρ coefficient	p-value	ρ coefficient	p-value
Gut dysbiosis score	0.054	0.313	0.073	0.170
Shannon index	0.003	0.956	-0.100	0.059
**Kendall correlation**	τ coefficient	p-value	τ coefficient	p-value
Gut dysbiosis score	0.037	0.296	0.049	0.167
Shannon index	0.004	0.918	-0.065	0.067
**Pearson correlation**	ρ coefficient	p-value	ρ coefficient	p-value
Gut dysbiosis score	0.057	0.288	0.077	0.149
Shannon index	-0.011	0.834	-0.076	0.152
**Multivariable-adjusted regression model**	β coefficient	p-value	β coefficient	p-value
Gut dysbiosis score	0.005	0.048	0.003	0.245

Shannon index	-0.020	0.297	-0.023	0.234
**HFD**				
**Spearman correlation**	ρ coefficient	p-value	ρ coefficient	p-value
Gut dysbiosis score	-0.063	0.235	0.006	0.917
Shannon index	0.066	0.214	-0.035	0.509
**Kendall correlation**	τ coefficient	p-value	τ coefficient	p-value
Gut dysbiosis score	-0.040	0.263	0.005	0.882
Shannon index	0.044	0.218	-0.024	0.504
**Pearson correlation**	ρ coefficient	p-value	ρ coefficient	p-value
Gut dysbiosis score	-0.055	0.297	0.031	0.566
Shannon index	0.074	0.166	-0.049	0.353
**Multivariable-adjusted regression model**	β coefficient	p-value	β coefficient	p-value
Gut dysbiosis score	-0.021	0.597	0.020	0.538
Shannon index	0.270	0.354	-0.367	0.196
**Vitamin Insufficiency**				
**Spearman correlation**	ρ coefficient	p-value	ρ coefficient	p-value
Gut dysbiosis score	-0.023	0.665	0.064	0.232
Shannon index	0.044	0.405	-0.059	0.267
**Kendall correlation**	τ coefficient	p-value	τ coefficient	p-value
Gut dysbiosis score	-0.015	0.691	0.046	0.221
Shannon index	0.031	0.403	-0.042	0.257
**Pearson correlation**	ρ coefficient	p-value	ρ coefficient	p-value
Gut dysbiosis score	-0.024	0.650	0.064	0.228
Shannon index	0.057	0.288	-0.050	0.349
**Multivariable-adjusted regression model**	β coefficient	p-value	β coefficient	p-value
Gut dysbiosis score	0.002	0.067	0.001	0.347
Shannon index	-0.004	0.634	-0.009	0.327

**Mineral insufficiency**				
**Spearman correlation**	ρ coefficient	p-value	ρ coefficient	p-value
Gut dysbiosis score	0.028	0.601	0.061	0.254
Shannon index	-0.042	0.430	-0.108	0.042
**Kendall correlation**	τ coefficient	p-value	τ coefficient	p-value
Gut dysbiosis score	0.019	0.621	0.041	0.272
Shannon index	-0.030	0.421	-0.078	0.039
**Pearson correlation**	ρ coefficient	p-value	ρ coefficient	p-value
Gut dysbiosis score	0.022	0.675	0.095	0.073
Shannon index	-0.021	0.691	-0.117	0.028
**Multivariable-adjusted regression model**	β coefficient	p-value	β coefficient	p-value
Gut dysbiosis score	0.002	0.099	0.002	0.114
Shannon index	-0.008	0.416	-0.021	0.028

We performed propensity score matching (1:1) to generate a balanced cohort and re-evaluated the associations within this matched sample. Statistical analysis was performed using Spearman, Kendall's τ, Pearson correlation two-tailed tests, and generalized linear models with adjustments of age, sex, GI conditions, and autistic symptoms. All reported exact p-values with significance defined as < 0.05.

Supplementary Table S4: Comparisons of demographic factors and dietary assessments between individuals with and without autism spectrum disorder (ASD).

	**ASD**<br>**N=462**	**Non-ASD**<br>**N=356**	**p value**
Age	8.00 (6.00-10.0)	9.00 (8.00-10.0)	<0.001
Male	395 (85.5%)	200 (56.2%)	<0.001
BMI	16.0 (14.6-18.1)	16.3 (14.8-18.7)	0.056
Family medical history	93 (20.1%)	19 (5.34%)	<0.001
Comorbidity (yes)	259 (56.1%)	0 (0.00%)	<0.001
Medication	150 (32.5%)	0 (0.00%)	<0.001
GI symptoms	92 (19.9%)	19 (5.34%)	<0.001
Bristol stool chart			0.05
type 1	25 (5.41%)	11 (3.09%)	
type 2	67 (14.5%)	45 (12.6%)	
type 3	100 (21.6%)	84 (23.6%)	
type 4	160 (34.6%)	146 (41.0%)	
type 5	29 (6.28%)	31 (8.71%)	
type 6	9 (1.95%)	1 (0.28%)	
type 7	1 (0.22%)	0 (0.00%)	
Autistic symptoms			
SRS_RRB	62.0 (55.0-71.0)	46.0 (43.0-50.0)	<0.001
SRS_SCI	68.0 (61.0-75.0)	53.0 (49.0-58.0)	<0.001
SEQ_hyper	2.07 (1.79-2.50)	1.57 (1.36-1.86)	<0.001
SEQ_hypo	1.67 (1.33-2.17)	1.33 (1.00-1.50)	<0.001
Eating behaviors (CEBQ)	0.17 (-0.42-0.79)	-0.26 (-0.82-0.37)	<0.001
Dietary variables			
AHEI	53.9 (20.6-81.4)	54.7 (26.8-86.3)	0.059
CCDI	74.3 (40.5-111)	79.0 (34.5-116)	<0.001
DII	0.295 (-4.09-4.19)	-0.220 (-4.27-4.30)	0.022
Sulfurdiet score	0.28 (-4.85-6.47)	-0.06 (-4.17-3.96)	<0.001
HFD	0.46 (0.20-0.69)	0.48 (0.26-0.76)	0.003
Mineral insufficiency	5.00 (1.00-12.0)	4.00 (0-12.0)	0.698
Vitamin insufficiency	7.00 (1.00-10.0)	7.00 (1.00-10.0)	0.861
Polysorbate-80	7.91 (0.36-40.9)	6.38 (0.20-33.9)	<0.001
Carboxymethylcellulouse	16.2 (0-90.6)	12.2 (0.08-55.0)	<0.001
Carrageenan	9.96 (0.28-57.6)	7.85 (0.48-40.6)	<0.001
Emulsifiers	35.1 (1.28-179)	27.4 (1.28-95.4)	<0.001
Protein (% of total energy)	14.9 (7.82-27.5)	16.5 (9.92-27.5)	<0.001
Fatcals (% of total energy)	30.6 (18.0-52.7)	29.7 (16.6-45.4)	0.600
Carbcals (% of total energy)	54.3 (17.9-73.9)	54.2 (29.4-72.1)	0.652
SFA (% of total energy)	10.1 (4.30-16.4)	10.3 (4.41-19.2)	<0.001
TFA (% of total energy)	0.17 (0.01-1.93)	0.15 (0-1.21)	0.181
PUFA (% of total energy)	4.77 (1.57-11.5)	4.08 (1.55-8.84)	<0.001
MUFA (% of total energy)	7.72 (0.61-21.8)	7.05 (0.767-15.1)	0.007

n3/n6	0.12 (0.04-0.42)	0.13 (0.07-0.29)	0.136
Monosaccharide (g/1000 kcal)	6.71 (0-55.8)	7.96 (0-35.2)	0.016
Disaccharide (g/1000 kcal)	3.77 (0-26.9)	4.91 (0-24.5)	0.004
Starch (g/1000 kcal)	76.7 (15.8-136)	74.2 (32.0-139)	0.033
Fiber (g/1000 kcal)	0.78 (0.09-11.30)	0.89 (0.19-4.51)	0.002
Added Sugar (g/1000kcal)	2.15 (0-50.9)	3.67 (0-27.8)	<0.001
Calcium (mg/1000 kcal)	267 (96.9-2170)	288 (78.8-972)	0.182
Phosphorus (mg/1000 kcal)	463 (189-1170)	495 (241-840)	<0.001
Sodium (mg/1000 kcal)	1190 (308-2700)	1290 (421-2770)	0.009
Potassium (mg/1000 kcal)	980 (464-2210)	1050 (507-2990)	<0.001
Magnesium (mg/1000 kcal)	102 (41.8-184)	107 (40.2-236)	0.001
Chromium (mg/1000 kcal)	2.69 (0.618-14.0)	2.69 (0.417-10.5)	0.884
Copper (mg/1000 kcal)	0.433 (0.15-1.53)	0.467 (0.18-1.49)	<0.001
Iodine (mcg/1000 kcal)	33.6 (0.28-195)	35.5 (1.54-193)	0.073
Iron (mg/1000 kcal)	5.10 (1.96-26.4)	5.45 (2.05-17.4)	0.048
Molybdenum (mcg/1000 kcal)	6.72 (0-33.1)	7.69 (0-47.8)	0.002
Selenium (mcg/1000 kcal)	44.5 (13.4-274)	48.7 (19.6-112)	0.014
Zinc (mg/1000 kcal)	4.10 (1.61-12.1)	4.34 (2.04-11.6)	0.020
Vitamin B1 (mg/1000 kcal)	0.43 (0.145-1.56)	0.437 (0.12-1.23)	0.993
Vitamin B2 (mg/1000 kcal)	0.57 (0.16-3.21)	0.57 (0.12-1.68)	0.198
Vitamin B3 (mg/1000 kcal)	6.32 (1.34-17.9)	6.68 (1.47-14.1)	0.014
Pantothenic acid (mg/1000 kcal)	2.01 (0.72-8.36)	2.10 (0.61-4.42)	0.045
Vitamin B6 (mg/1000 kcal)	0.31 (0.06-1.24)	0.30 (0.08-1.11)	0.614
Vitamin B12 (mg/1000 kcal)	0.292 (0-2.96)	0.328 (0-4.08)	0.662
Biotin (mcg/1000 kcal)	9.21 (1.26-57.9)	10.2 (1.22-43.3)	0.003
Vitamin C (mg/1000 kcal)	40.3 (1.65-254)	43.3 (0.94-235)	0.088
Vitamin D (mcg/1000 kcal)	1.62 (0.12-11.4)	1.77 (0.04-8.25)	0.423
Folate (mcg/1000 kcal)	89.1 (17.4-323)	96.2 (11.2-297)	<0.001
Vitamin K (mcg/1000 kcal)	29.5 (2.32-342)	34.3 (0.01-350)	0.042
Caffeine (mg/1000 kcal)	0.0262 (0-11.8)	0.956 (0-23.4)	<0.001
Chlorophyll (mg/1000 kcal)	128 (25.8-453)	141 (42.0-747)	<0.001
Fruit (g/1000 kcal)	113 (0-710)	145 (0-1490)	<0.001
Vegetable (g/1000 kcal)	145 (0-1050)	194 (0-1830)	<0.001
Sugarjam (g/1000 kcal)	4.65 (0-175)	4.29 (0-77.1)	0.105
Processed meat (g/1000 kcal)	18.8 (0-266)	18.0 (0-163)	0.265
Fried food (g/1000 kcal)	37.3 (0-180)	40.4 (0-178)	0.741
Dark green vegetables (g/1000 kcal)	20.0 (0-166)	28.6 (0-480)	<0.001
Dairy (g/1000 kcal)	66.5 (0-1000)	81.1 (0-773)	0.420
Beans (g/1000 kcal)	15.4 (0-271)	22.1 (0-196)	0.015

Macronutrients were presented as percentages of total energy intake. Group comparisons were performed using Kruskal-Wallis rank sum tests for continuous variables and Pearson's Chi-squared tests were used for categorical variables. All reported exact p-values are two-tailed testing. SRS, the Social Responsiveness Scale; SEQ, the Sensory experiences questionnaire; CCDI, the Chinese Children Healthy Dietary Index; AHEI, Alternative Healthy Eating Index; DII, Dietary Inflammatory Index; HFD, healthy food diversity index; SFA, saturated fatty acids;

MUFA, monounsaturated fatty acids; PUFA, polyunsaturated fatty acids; TFA, trans-fatty acids.
