# Supplementary material for: Distinct diet-microbiome associations in autism spectrum disorder
Source: Nat Commun. 2025 Dec 31;17:3109. doi: 10.1038/s41467-025-67711-7 (PMC13039903; doi:10.1038/s41467-025-67711-7)
Supplement: Supplementary file 2 — Description of Additional Supplementary Files [file 41467_2025_67711_MOESM2_ESM.docx]

**Description of Additional** **Supplementary Files**

**Supplementary Data 1:** Effect sizes (MaAsLin2 coefficients) and adjusted q-values for the diet-microbiome associations in children with and without autism spectrum disorder (ASD). Generated for Figure 2.

**Supplementary Data 2:** Sensitivity analysis for the diet-microbiome associations for autism spectrum disorder (ASD)-specific species in the 1:1 matched-ASD group (n=356). All dietary indices were re-analyzed in the 1:1 matched ASD cohort using the same MaAsLin2 method, with adjustment for multiple covariates. Significant p-values were corrected for multiple comparisons using the false discovery rate approach and reported as adjusted q-values. Supplemented for Figure 2.

**Supplementary Data 3:** Effect sizes and adjusted q-values for the diet-microbiome associations incorporated with the interaction term (diet × ASD) across the study population. Generated for Supplementary Fig. 4.

**Supplementary Data 4:** The list of microbial species most affected by diet, categorized separately for the general population and for children with or without autism spectrum disorder (ASD). Generated for Fig. 5A.

**Supplementary Data 5:** Alterations in microbial species associated with four key dietary factors in children with and without autism spectrum disorder (ASD). The low intake group is used as the reference compared to the high intake group. Generated for Fig. 3A and Supplementary Fig. S7.

**Supplementary Data 6:** Sensitivity analysis for the alterations of microbial species associated with diet, and diet-by-autism spectrum disorder (ASD) interaction after 1:1-sample size matching. The low intake group is used as the reference compared to the high intake group. Supplemented for the sensitivity analysis of Fig. 3A and Supplementary Fig. S7.

**Supplementary Data 7:** Alterations in microbial functional pathways associated with four key dietary factors in children with and without autism spectrum disorder (ASD). The low intake group is used as the reference compared to the high intake group. Associations between microbial functionality and dietary factors were assessed using MaAsLin2, while controlling for age, sex, and GI conditions in children with autism spectrum disorder (ASD) and those without. Significant p-values were corrected for multiple comparisons using the false discovery rate approach and reported as adjusted q-values. Generated for Fig. 3B.

**Supplementary Data 8:** The mediating role of the gut microbiome in the associations between dietary metrics and core symptoms of children with autism spectrum disorder (ASD). The Average Causal Mediation Effect (ACME) with 95% confidence interval (CI) that did not include zero, was considered evidence of a potential mediation effect. Significant p-values for mediations were corrected for multiple comparisons using the false discovery rate approach and reported as adjusted q-values. SRS, the Social Responsiveness Scale; SEQ, the Sensory experiences questionnaire; CCDI, the Chinese Children Healthy Dietary Index; AHEI, Alternative Healthy Eating Index; DII, Dietary Inflammatory Index; HFD, healthy food diversity index; SFA, saturated fatty acids; MUFA, monounsaturated fatty acids; PUFA, polyunsaturated fatty acids; TFA, trans-fatty acids. Generated for Supplementary Fig. S8.
