# Supplementary material for: Distinct diet-microbiome associations in autism spectrum disorder
Source: Nat Commun. 2025 Dec 31;17:3109. doi: 10.1038/s41467-025-67711-7 (PMC13039903; doi:10.1038/s41467-025-67711-7)
Supplement: Supplementary file 4 — Reporting Summary [file 41467_2025_67711_MOESM4_ESM.pdf]

nature portfolio

Corresponding author(s): Siew C Ng

Last updated by author(s): Nov 29, 2025

Reporting Summary

Nature Portfolio wishes to improve the reproducibility of the work that we publish. This form provides structure for consistency and transparency in reporting. For further information on Nature Portfolio policies, see our Editorial Policies and the Editorial Policy Checklist.

Statistics

For all statistical analyses, confirm that the following items are present in the figure legend, table legend, main text, or Methods section.

n/a	Confirmed	
☐	☒	The exact sample size (*n*) for each experimental group/condition, given as a discrete number and unit of measurement
☐	☒	A statement on whether measurements were taken from distinct samples or whether the same sample was measured repeatedly
☐	☒	The statistical test(s) used AND whether they are one- or two-sided<br>*Only common tests should be described solely by name; describe more complex techniques in the Methods section.*
☐	☒	A description of all covariates tested
☐	☒	A description of any assumptions or corrections, such as tests of normality and adjustment for multiple comparisons
☐	☒	A full description of the statistical parameters including central tendency (e.g. means) or other basic estimates (e.g. regression coefficient) AND variation (e.g. standard deviation) or associated estimates of uncertainty (e.g. confidence intervals)
☐	☒	For null hypothesis testing, the test statistic (e.g. *F*, *t*, *r*) with confidence intervals, effect sizes, degrees of freedom and *P* value noted<br>*Give P values as exact values whenever suitable.*
☒	☐	For Bayesian analysis, information on the choice of priors and Markov chain Monte Carlo settings
☒	☐	For hierarchical and complex designs, identification of the appropriate level for tests and full reporting of outcomes
☐	☒	Estimates of effect sizes (e.g. Cohen's *d*, Pearson's *r*), indicating how they were calculated

Our web collection on statistics for biologists contains articles on many of the points above.

Software and code

Policy information about availability of computer code

Data collection	For full details see Methods; Trimmomatic (v39), Bowie2 (v2.4.2), KneadData (v0.6), Kraken 2 (v2.1.2), Bracken (v2.5.0), HUMAnN (v3.0), GNU parallel (v3.0) were used to process the microbiome sequencing data. Food Processor Nutrition Analysis and Fitness Software (version 8.0, ESHA Research) was used to collect nutrition intake.
Data analysis	All software used were from publicly available sources. R packages including compositions (v2.0-5), phyloseq (v1.24.2), vegan (v2.6-4), RF (v2.2), pROC (v1.18.5), ggpubr (v0.6.0), MaAsLin2 (v1.4.0), Sparcc (v4.4.1), iGraph (v4.4.1) were used to analyze and visualize the microbiome profiling data. Open source code for MDiNE model is available at https://github.com/kevinmcgregor/mdine. A 1:1 nearest-neighbor matching algorithm was used via the MatchIt(v3.7.2) package in R, matching on age, sex, and GI conditions.

For manuscripts utilizing custom algorithms or software that are central to the research but not yet described in published literature, software must be made available to editors and reviewers. We strongly encourage code deposition in a community repository (e.g. GitHub). See the Nature Portfolio guidelines for submitting code & software for further information.

Data

Policy information about availability of data

All manuscripts must include a data availability statement. This statement should provide the following information, where applicable:

- Accession codes, unique identifiers, or web links for publicly available datasets
- A description of any restrictions on data availability
- For clinical datasets or third party data, please ensure that the statement adheres to our policy

The metagenomic sequencing data used in this study have been published before and were available in the NCBI Sequence Read Archive database under accession code PRJNA943687 (https://www.ncbi.nlm.nih.gov/bioproject/?term=PRJNA943687) 8. Processed dietary and microbial data are deposited in Zenodo (https://zenodo.org/) under the identifier No.17730818 55. The full results of the diet-microbiome association analyses, including all exact p-values, are provided in the Source Data file. Source data are provided with this paper. Participant metadata cannot be made publicly available via repositories as outlined in the patient consent form to protect participant privacy. Requests for sharing metadata, including dietary profiles can be submitted with a written proposal to corresponding author (Prof. Siew C. Ng) at siewchienng@cuhk.edu.hk. The proposal should detail the intended use of the data. The data management team composed of scientists and clinicians will review these requests based on scientific merit and ethical considerations, including patient consent, to avoid any misuse or misinterpretation. Data sharing will be undertaken if the proposed projects have a sound scientific rationale or potential patient benefit. Data recipients are required to enter formal data sharing agreement, which describes the conditions for release and requirements for data transfer, storage, archiving, and publication. Since the data management meeting is held monthly, please anticipate a response within two working months. Data access is typically granted for 12 months under a Data Use Agreement that prohibits participant re-identification and third-party data transfer.

Research involving human participants, their data, or biological material

Policy information about studies with human participants or human data. See also policy information about sex, gender (identity/presentation), and sexual orientation and race, ethnicity and racism.

Reporting on sex and gender	A total of 818 children (ASD=462 and non-ASD=356, 27.3% females, age range: 3-12 years) of Chinese ethnicity with compete dietary and gut metagenomics data were included in the current study. The information was collected during clinical interviews and verified from the clinical management system of hospital authority in Hong Kong.
Reporting on race, ethnicity, or other socially relevant groupings	No socially relevant grouping were involved in this study.
Population characteristics	We reported this information in Table 1, Table S1, and Table S4. Dietary profiling in a cohort of 818 children revealed that individuals with poorer dietary patterns—categorized by tertiles of the Chinese Children Healthy Dietary Index (CCDI)—exhibited more severe autistic symptoms, increased medication use, GI complications, and challenges across multiple eating behaviors, including desire to drink, food enjoyment, fussiness, and satiety responsiveness (all $p < 0.05$, Table 1). Other phenotype data included parental socioeconomic status, BMI, Bristol Stool Form Score. Further dietary comparisons identified nutritional insufficiency and low dietary quality across multiple metrics, including the Alternative Healthy Eating Index (AHEI), Dietary Inflammatory Index (DII), sulfur-diet score, and healthy food diversity (HFD) index, alongside elevated polysorbate-80 (P80) exposure were observed. Table S9 provides additional information in a case-control manner, demonstrating the dietary data distribution and range of behavioral assessments.
Recruitment	We initiated a prospective case-control study in Hong Kong, aiming at investigating the correlation between gut microbiota composition and autism symptoms and severity over time, as well as the validation of previously identified microbial markers of ASD considering genetics and other confounding factors including ASD-related symptoms and dietary information. Children with ASD were diagnosed by psychiatrists according to the fifth edition of the Diagnostic and Statistical Manual of Mental Disorders (DSM-5) and were recruited from the Child and Adolescent Psychiatric Clinic of the New Territory East Cluster (NTEC) of the Hospital Authority in Hong Kong. Non-autistic children, without first-degree relatives diagnosed with autism by screening negative on the Autism-Spectrum Quotient-10 (AQ-10) and absence of psychiatric disorder according to the DISC-5, were recruited from the community during the same period. Both groups included children under the age of 12, alongside exclusion criteria that comprised a known history of intellectual disability, psychosis, depression, and neurological disorders.
Ethics oversight	The study protocol adhered to the principles outlined in the Declaration of Helsinki and received ethics approval from the Joint Committee on Clinical Research Ethics, CUHK-New Territories East Hospital Cluster (CUHK-NTEC CRE, Ref.: 2021.550). Written informed consent was obtained from the parents or caregivers of the participants.

Note that full information on the approval of the study protocol must also be provided in the manuscript.

Field-specific reporting

Please select the one below that is the best fit for your research. If you are not sure, read the appropriate sections before making your selection.

[x] Life sciences [ ] Behavioural & social sciences [ ] Ecological, evolutionary & environmental sciences

For a reference copy of the document with all sections, see nature.com/documents/nr-reporting-summary-flat.pdf

Life sciences study design

All studies must disclose on these points even when the disclosure is negative.

Sample size	No statistical methods were used to pre-determine sample sizes, but our sample sizes are similar to hose reported in previous publications.<br>Liu NN, et al. Multi-kingdom microbiota analyses identify bacterial-fungal interactions and biomarkers of colorectal cancer across cohorts. Nat Microbiol. 2022 Feb;7(2):238-250.<br>Tito, R.Y., et al. Microbiome confounders and quantitative profiling challenge predicted microbial targets in colorectal cancer development. Nat Med 30, 1339–1348 (2024).
Data exclusions	Both groups included children under the age of 12, alongside exclusion criteria that comprised a known history of intellectual disability, psychosis, depression, and neurological disorders. Samples were all successfully sequenced and passed the quality assessment (read depth > 10 million). Subjects with incomplete dietary reports were excluded resulting in a total of 818 children (ASD=462 and non-ASD=356).
Replication	To ensure reliable performance estimates, we applied the rfcv function from the 'RF' package using a repeated 10-fold cross-validation strategy (ntree 1000, using default mtry). The mean AUC value was calculated accordingly for visualization of results.<br>We applied MDiNE model fitted by Hamiltonian Monte Carlo method (iterations=1000) to estimate changes in the fecal microbial network topology within the groups.
Randomization	The conventional randomization (as used in clinical trials or interventional studies) was not relevant for this study because this study not include any interventions.
Blinding	The conventional blinding (as used in clinical trials or interventional studies) was not relevant for this study because this study not include any interventions.

Reporting for specific materials, systems and methods

We require information from authors about some types of materials, experimental systems and methods used in many studies. Here, indicate whether each material, system or method listed is relevant to your study. If you are not sure if a list item applies to your research, read the appropriate section before selecting a response.

Materials & experimental systems

n/a	Involved in the study
☒	☐ Antibodies
☒	☐ Eukaryotic cell lines
☒	☐ Palaeontology and archaeology
☒	☐ Animals and other organisms
☒	☐ Clinical data
☒	☐ Dual use research of concern
☒	☐ Plants

Methods

n/a	Involved in the study
☒	☐ ChIP-seq
☒	☐ Flow cytometry
☒	☐ MRI-based neuroimaging

Plants

Seed stocks	*Report on the source of all seed stocks or other plant material used. If applicable, state the seed stock centre and catalogue number. If plant specimens were collected from the field, describe the collection location, date and sampling procedures.*
Novel plant genotypes	*Describe the methods by which all novel plant genotypes were produced. This includes those generated by transgenic approaches, gene editing, chemical/radiation-based mutagenesis and hybridization. For transgenic lines, describe the transformation method, the number of independent lines analyzed and the generation upon which experiments were performed. For gene-edited lines, describe the editor used, the endogenous sequence targeted for editing, the targeting guide RNA sequence (if applicable) and how the editor was applied.*
Authentication	*Describe any authentication procedures for each seed stock used or novel genotype generated. Describe any experiments used to assess the effect of a mutation and, where applicable, how potential secondary effects (e.g. second site T-DNA insertions, mosiacism, off-target gene editing) were examined.*
